# Practice of non-pharmaceutical interventions against COVID-19 and reduction of the risk of influenza-like illness: a cross-sectional population-based study

**DOI:** 10.1186/s40545-022-00450-y

**Published:** 2022-08-30

**Authors:** Dalal Youssef, Ola Issa, Maysaloun Kanso, Janet Youssef, Linda Abou-Abbas, Edmond Abboud

**Affiliations:** 1 Research Center Bordeaux Population Health, Institut de Santé Publique, d’épidémiologie et de Développement (ISPED), Bordeaux University, Bordeaux, France; 2 Clinical Trial Program, Ministry of Public Health, Beirut, Lebanon; 3 Preventive Medicine Department, Ministry of Public Health, Beirut, Lebanon; 4 Ministry of Public Health, Beirut, Lebanon; 5 Al Zahraa Hospital University Medical Center, Beirut, Lebanon; 6 Neurosciences Research Center, Faculty of Medical Sciences, Lebanese University, Beirut, Lebanon

**Keywords:** Precautionary measures, COVID-19, Influenza-like illness, Reduction, Non-pharmaceutical interventions

## Abstract

**Introduction:**

While the widespread implementation of the non-pharmaceutical interventions was intended to contain the COVID-19 pandemic, such measures could be also effective in limiting the spread of other respiratory infections. This study aimed to examine the association between the implementation of personal protective measures and the occurrence of influenza-like illnesses (ILI) in the general population.

**Methods:**

An online retrospective cross-sectional observational study was conducted in April 2021 to assess cases of ILI among Lebanese adults aged 18 years and above, from all Lebanese governorates during the 2020–2021 flu season. Data were collected using a convenience sampling method. In addition to their socio-demographic information, participants were asked about their frequency of implementing personal protective measures and if they have experienced symptoms of ILI in the previous 6 months. The overall score of the personal protective measures was computed. Multivariable logistic regression was performed to examine the association between participants’ level of adoption of personal protective measures against COVID-19 and the occurrence of ILI.

**Results:**

Among the 1019 Lebanese adults participating in this study, 352 (34.54%) of them reported symptoms of ILI between October 2020 and March 2021. Lebanese adults who wore their facemasks frequently or always were less likely to suffer from symptoms of ILI than others who did not wear the mask (aOR = 0.452, 95% CI = 0.349–0.693, *p* < 0.001). Similarly, adults who adopt the following protective measures washing hands (aOR = 0.608, 95% CI = 0.524–0.922, *p* < 0.001), respecting cough etiquette (aOR = 0.763, 95% CI = 0.598–0.918, *p* < 0.001), disinfecting surface (aOR = 0.892, 95% CI = 0.632–0.911, *p* = 0.012), avoiding crowded places (aOR = 0.739, 95% CI = 0.688–0.903, *p* = 0.049), respecting physical distancing (aOR = 0.646, 95% CI = 0.482–0.833, *p* = 0.031) on a regular basis (frequently/always) were less likely to report symptoms of influenza-like illnesses when compared with those who did not adhere at all to these measures.

**Conclusion:**

Our study highlighted the potential of personal protective measures against COVID-19 in reducing the transmission of respiratory infections such as ILI. Such findings might be invested during influenza season, particularly among groups at high risk of developing severe complications. Exploring trends detected by the national severe acute respiratory infection surveillance system is recommended to confirm the utility of these measures.

## Background

The severe acute respiratory syndrome coronavirus-2 (SARS-CoV-2) is a novel virus that emerged in China in late 2019 and then turned into a worldwide disaster [1]. Given the novelty of the causative virus, there was a lack of available pharmaceutical options to fight it such as vaccines and specific antiviral treatments [2]. At this point, non-pharmaceutical interventions (NPIs) seem like the merely available option, gaining, therefore, prominence over other methods [3]. To curtail the virus transmission and reduce mortality, several preventive measures to protect the communities and individuals through NPIs were recommended by the World Health Organization (WHO) [4]. Moreover, a variety of health policies and large-scale public health measures have been implemented proactively by governments worldwide [5] to contain the ongoing COVID-19 pandemic and to gain time awaiting the availability of pharmaceutical interventions. At the country level, these protective measures included limiting or banning international travel, stringent lockdown, remote work, and cancellation of public events. At the personal level, several health-related behaviors were recommended. The latter encompassed mandatory public use of facemask, regular hand hygiene, compliance to cough etiquette, keeping physical distancing, staying at home when feeling sick, disinfecting touched surfaces and objects avoiding the 3Cs such as crowded places and social gatherings, close contacts, and closed spaces [4]. In this context, the findings of various studies have supported the effectiveness of wearing facemasks, protecting the eyes, physical distancing [6], and hand hygiene [7] in impeding the transmission of SARS-CoV-2. A recent systematic review and meta-analysis disclosed that keeping a physical distance of one meter or more can considerably lower the risk of viral transmission [6]. Similarly, mask and eye protection use also ensued a large decrease in the risk of infection (mask use: aOR 0.15; eye protection: aOR 0.22) [6]. Moreover, the use of masks by all residents was a key component to successfully combating COVID-19 and may have reduced fear and anxiety [8–10]. It is worth mentioning that evidence also supported the potential role of limiting contact measure with COVID-19 case, during his or her incubation period by reducing the frequency and the duration of contact, in reducing the average number of individuals to whom the virus was transmitted [11]. Even with these procedures in play, reduced and unequal access to health care worldwide was noted due to the overstressing of health systems and the economic burden caused by the pandemic [12].

While this widespread implementation of the above-mentioned preventive measures was intended to mitigate and contain the COVID-19 pandemic, such procedures could be also effective in limiting the spread of other respiratory illnesses such as seasonal influenza, outpatient pneumonia, and severe acute respiratory infection (SARI) and flu-like illnesses [13, 14]. This could be anticipated since SARS-CoV-2 and other viruses causing acute respiratory infections shared similar transmission routes and are spread mainly by respiratory droplets. Despite the paucity of data related to the effectiveness of these measures in preventing community transmission of influenza-like illnesses [15, 16], Olsen et al. reported a sharp decline in influenza activity and influenza-like illnesses after the implementation of the above-mentioned measures [15]. In addition, several reports have shown a decrease in the number of influenza cases during the 2019–2020 influenza season. This was supported by the influenza data reported to the WHO through the FluNet platform in 2021, where a decline in influenza-positive results and outpatient visits for influenza-like illnesses [17] was recorded in the majority of countries and regions in the Southern, Northern Hemispheres, and temperate zones. A study conducted in New Zealand reported that the incidence of influenza decreased 79-fold during the post-lockdown period in addition to a significant reduction in the incidence of other respiratory viruses in comparison with the same period in the previous year [18]. Similar findings were reported in a study conducted in Japan where a decrease in the number of people infected with the influenza virus in 2020 was reported, compared to the past year [19].

In Lebanon, the first case of COVID-19 was confirmed on 21 February 2020 [20]. Since the early phase of the pandemic, Lebanon has been pre-emptive in responding to COVID-19 by strengthening and maintaining its national capacities required under the International Health Regulations (IHR 2005) [21]. A National Committee for COVID-19 (NCC) was established to lead and run the COVID-19 national preparedness and response using a holistic approach involving all stakeholders including proactive measures to prevent, to control the spread of COVID-19. Similar to other countries, Lebanon has promoted health-related behaviors such as hand-washing and physical distancing to protect communities and individuals from the transmission of COVID-19. Of note, acquiring such behavioral insights will be essential to boost and encourage compliance with recommended practices and managing disease transmission. On March 14, 2020, with the rise of COVID-19 cases to 99, Lebanon declared a state of health emergency and the government imposed a 2-week “lockdown” on people’s movements as part of the country’s efforts to slow the spread of the virus [22]. Since the acceptance and adoption of such health behaviors by community members during COVID-19 is associated with communication, the lockdown was accompanied by a high level of COVID-19 risk communications and an upgrade of preventive measures. Such behavioral practice required that effective operational strategies are put into place. In addition, it is also associated with the perceived risk of COVID-19, the level of knowledge, and the perceived level of effectiveness of such health-related behaviors among community members [23]. Briefly, although these customary strategies may have benefits based on current evidence during the COVID-19 pandemic, their effectiveness on other respiratory infections apart from SARS-CoV-2 remains largely unclear in the Lebanese context, similar to other countries. Therefore, it is of great interest to explore the potential of the protective behavioral practices adopted by Lebanese adults in limiting the spread of influenza-like illnesses among the Lebanese adult population.

This study aimed to examine the association between the implementation of personal precautionary measures and the cases of influenza-like illnesses in the general population.

## Methods

### Study tool and design

A retrospective cross-sectional observational study was carried out in April 2021 to assess cases of influenza-like illnesses among Lebanese adults during the 2020–2021 flu season (from October 2020 and the end of March 2021). Data were collected through an online survey using a convenience sampling method. As the Lebanese government recommended the public to minimalize face-to-face interaction, potential respondents were electronically invited to participate. To minimize selection bias related to the convenience sampling technique used and to ensure better representativeness of the Lebanese population in terms of age, gender, and residence (Bekaa, Baalbeck-Hermel, Mount-Lebanon, Beirut, North, Akkar, South, and Nabatyeh), a weighting procedure was adopted using predetermined target figures. The latter helped in aligning the sample distribution with the population for the above-mentioned variables.

### Questionnaire development

A review of the literature was conducted to list available resources on recommended NPIs during COVID-19 with a special focus on personal health-related behaviors [24–27]. A 40-item structured questionnaire was initially developed and designed by the authors to cover important aspects of adopted NPIs among the Lebanese population which consisted of closed-ended questions. The internal consistency reliability of the English version of the questionnaire was estimated using Cronbach’s alpha where its value α ≥ 0.70 was considered satisfactory [28]. An expert panel was appointed including eight members (two epidemiologists, two infectious disease specialists, two physicians, and two lay experts) to assess the content validity of the questionnaire and to confirm whether the instrument adequately or exhaustively contains all the items necessary to cover the study objective. Experts were defined as individuals who had a good understanding of the infection preventive measures and who worked in the field of infectious diseases. Content validity of the questionnaire was assessed using the experts' quantitative and qualitative viewpoints on the relevancy or representativeness and clarity of the items to measure the construct operationally‏ defined by these items. Each question of the questionnaire was evaluated by rating a) its relevance to the instrument’s aim and b) its understandability/clarity on a 4-point scale (1 = not relevant/not clear/4 = highly relevant/clear). In addition, the experts were asked to evaluate whether the items covered all important aspects or if there were missing components. The content validity index (CVI) was calculated both at the item level (I-CVI) and scale level (S-CVI) for all the attributes. On the item level, 88.3% of the ratings had a CVI greater than or equal to 0.78 based on the quantitative approach used by experts. None of the ratings were below 0.50. Therefore, all the 40 items were retained and the panel of experts considered the instrument appropriate and judged that the questionnaire had good content validity.

Then, the original 40-item version of the questionnaire was forward translated from English to the Arabic language by an epidemiologist whose mother tongue is Arabic and who was also proficient in English. Then, the translated version was back-translated by another epidemiologist who was a native speaker of the English language. A committee of experts was composed to verify the translation and matched the back-translated English questionnaire with the original scale version to detect inconsistencies. The questionnaire was assessed for the clarity of the questions and the accuracy of the domains. Any suggested linguistic change that should be made to the translated version was resolved by consensus.

The pre-final version of the questionnaire was also pretested on a convenience sample of 20 male and 20 female Lebanese adults (*n* = 40) to ensure survey flow, readability, clarity of interpretation, comprehension of instructions, and validity of responses. Minor modifications in terms of readability and clarity were made to the questionnaire based on the feedback of the respondents of the pre-test [29]. This included the adaptation of potentially misleading items and ambiguous words revealed during the pilot testing to the lay language leading to the production of the final Arabic version of the questionnaire. Its reliability was also checked, and the Cronbach Alpha value was calculated indicating good reliability (α = 0.79). The average time for completing the self-administered survey was 7 min.

The final version of the questionnaire consisted of four main sections with closed-ended questions:

1. The baseline characteristics of the study participants section included information about age, gender, marital status, educational level, urbanicity, working status, health status, underlying health conditions, current smoking status, and health coverage of the participants. Surveyed adults were also asked whether they had a previous history of COVID-19 infection and if they have tested positive for COVID-19 during the current influenza season (October 2020 to March 2021).
2. Vaccination status: Participants were asked if they have received the influenza vaccine for the current season and if they are being vaccinated against COVID-19.
3. Influenza-like illness section: Participants were asked to answer on a yes or no basis if they experienced symptoms of influenza-like illnesses (ILI) in the previous months. In this section, ILI symptoms were defined as an acute respiratory infection with an onset within 10 days, fever of > 38 °C, and persistent cough in the absence of an alternative causative agent [30, 31]. Adults who reported symptoms of fever, sore throat, and persistent cough and did not receive any confirmatory tests were regarded as having symptoms of influenza-like illnesses, consistent with the WHO’s case definition of influenza-like illnesses.
4. Personal protective measures: Participants enrolled were asked about their frequency of adopting the following personal protective measures: wearing facemasks, hand hygiene, surface disinfection, physical distancing, and avoiding crowded places. Participants reported their frequency of implementing the measures using a 5-Likert scale (never = 1, rarely = 2, sometimes = 3, frequently = 4, always = 5).

### Study participants

All Arab-speaking Lebanese adults aged 18 years or above from all the eight Lebanese governorates (Bekaa, Baalbeck-Hermel, Beirut, Mount Lebanon, North, Akkar, Nabatieh, South) having internet access and literacy and who gave their consent to participate were eligible for participation in the study. The study excluded Lebanese adults who did not speak Arabic, foreign participants (adults from other nationalities living in Lebanon), those suffering from comprehension problems, and those who do not have internet access or internet literacy. As influenza and SARS-CoV-2 are symptomatically indistinguishable, individuals with positive SARS-CoV-2 tests or those who were tested negative for COVID-19 but had contact with COVID-19 cases were also excluded from the study.

### Sample size calculation

Assuming that around three million adults reside in Lebanon [32], a 95% confidence level was used and an absolute error was estimated to be 5%. All previous information was used to calculate the sample size for this study using the Raosoft sample size calculator which yielded the least required sample size of 385 participants [33]. A rough estimate was made by multiplying the calculated sample size by 2.65 times, resulting in a final sample size of 1019 individuals, which reduced sampling error and increased study power.

### Data collection

Potential participants were recruited through social media platforms, institutional and university groups. The link of the online questionnaire developed using a Google form included a summary of the study’s background, its objective, and some instructions to the respondents for facilitating the completion of the questionnaire. Participants were screened based on the responses given in the baseline questionnaire to determine whether they met the study's inclusion criteria. No reward was given to respondents for their participation.

## Ethical considerations

Given the online nature of the study, electronic informed consent was obtained for each participant. Respondents were reassured their participation is solicited, yet strictly voluntary. They were free to withdraw at any time without any penalty. All information was gathered anonymously and handled confidentially. As individual participants cannot be identified based on the presented material, this study caused no plausible harm or stigma to participants and there will be no foreseeable risks for this study and no direct benefit as well. However, the information obtained may help in providing evidence about the effectiveness of personal protective behavior in decreasing ILI. The study design respected the participant's confidentiality and assured adequate protection of study participants, and neither included clinical data about patients nor configured itself as a clinical trial. This study was exempted from ethical approval by the Ministry of Public Health after a review of the study protocol.

### Statistical analysis

All descriptive and comparative analyses were performed using the Statistical Package for the Social Sciences to IBM SPSS 24. Categorical variables with ordinal response scales were grouped according to the frequency of each personal protective measure (wearing facemasks, washing hands, cough etiquette, physical distancing, avoiding crowded places, and disinfecting surfaces) into three groups. A value of 0 was assigned for participants who never or rarely applied each specific measure. A value of 1 was assigned for the “sometimes” option and the value of 2 was assigned for the “frequently” or “always” options [27]. The overall score of the personal protective measure was equal to the sum of each protective measure for each participant and the latter ranged between 0 and 12 where these 2 values represented the lowest and highest scores, respectively. The frequency of adoption of personal protective measures among Lebanese adults was compared between individuals who had influenza-like symptoms and those who did not. Multivariable logistic regression was carried out on the significant variables in the bivariate analyses (Chi-squared test with *p*-value < 0.2), to identify the factors associated with the occurrence of influenza-like illnesses. The use of *p* < 0.2 as the stopping rule to identify the set of covariates in the bivariate model to be included in the multivariable model might not provide an optimal variable selection for the covariates, although previous studies have provided a strong recommendation for using *p*-values in the range of 0.15–0.20. The first regression analysis included covariates such as gender, age, educational level, residence, health status, current smoking status, and influenza vaccine intake (*p*-value less than 0.2 when running bivariate analysis) in addition to the level of implementation of each personal protective measure. As per the result of the first regression, the covariates that were found significantly associated with the occurrence of ILI were controlled and the analysis was rerun with the personal protective measures against COVID-19 (wearing facemasks, hand hygiene, physical distancing, surface disinfection, and avoiding crowded places) set as the covariates (block 2). Significance was set as *p*-value < 0.05.

## Results

### Baseline characteristics of the participants

A total of 1019 Lebanese adults who met the inclusion criteria have agreed to participate in this survey. Table 1 displays the baseline information of the participants. Majority of the respondents were females (53.4%), married (81.1%), aged between 30 to 49 years, and had a higher educational level than secondary level (66.7%). Most of them were residing in urban areas (66.1%), particularly in Mount Lebanon province (26.7%). The majority of participants (83.3%) ranked their health status as good or above. On the other hand, around 40% of them were current smokers.

**Table 1:** Baseline characteristics of the study population

	*N*	%
Gender		
Male	475	46.60
Female	544	53.40
Age groups (years)		
18–29	290	28.50
30–49	555	54.50
50 and above	174	17.10
Marital status		
Single	152	14.90
Married	826	81.10
Other (divorced, widowed)	41	4
Educational level		
Secondary or below	339	33.30
More than secondary (University, Master, etc.)	680	66.70
Urbanicity		
Rural	345	33.90
Urban	674	66.10
Province		
Great Bekaa	176	17.30
North and Akkar	211	20.70
South and Nabatyeh	150	14.70
Mount Lebanon	272	26.70
Beirut	210	20.60
Working status		
No	174	17.10
Yes	845	82.90
Profile		
Student	149	14.60
Worker	822	80.70
Other (retired, housewife, etc.)	48	4.70
Perceived health status		
Fair or below	170	16.70
Good and above	849	83.30
Underlying health condition		
No	853	83.70
Yes	166	16.30
Current smoking status (shisha or cigarette)		
Non-smoker	613	60.20
Smoker	406	39.80
Total	1019	100

*N* frequency, %: percentage

### Influenza-like illness among participants

**Fig. 1:**
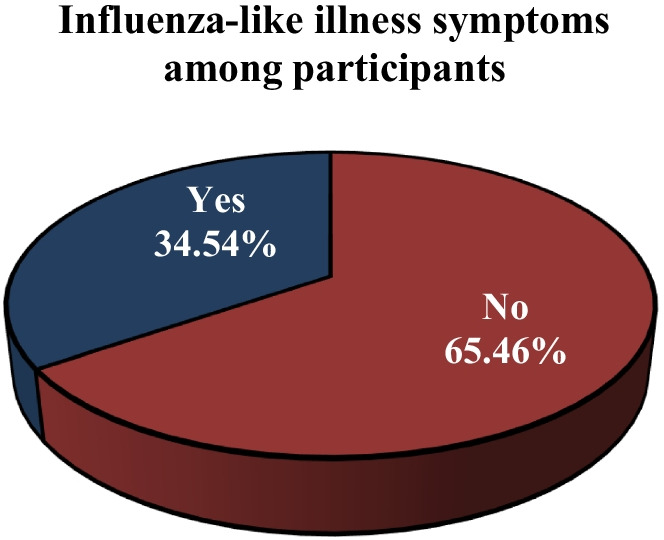
Occurrence of influenza-like illness among participants

**Fig. 2:**
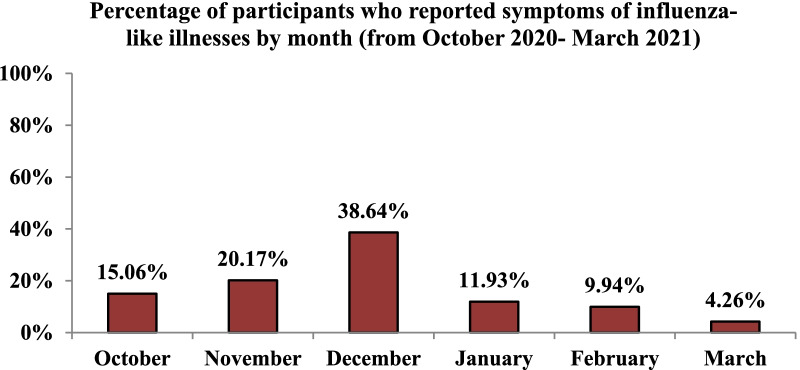
Influenza-like illness cases by month of occurrence

Between October 2020 and March 2021, a total of 352 (34.54%) individuals experienced symptoms of influenza-like illness, as indicated in Fig. 1. The number of ILI cases rapidly climbed from October (15.06%) to December (38.64%), when the majority of ILI symptoms appeared. Then, throughout the next few months, the frequency of cases declined, reaching its lowest point in March (4.26%) (Fig. 2).

### Implementation of personal protective measures by participants

Table 2 displays the frequency of implementation of each personal protective measure by Lebanese adults. We found that participants who did not have symptoms of influenza-like illnesses reported a higher level of implementation of all the personal protective measures than those who experienced symptoms. However, around 20% of those who showed ILI symptoms did not adhere or rarely adhere to the majority of protective measures.

**Table 2:** Frequency of each recommended personal protective measure as applied by participants

	*N*	Never/rarely	Sometimes	Frequently/always
		*n* (%)	*n* (%)	*n* (%)
Participants without influenza-like illness	667			
Wearing facemask	667	7 (1%)	158 (23.7%)	502 (75.3%)
Hand hygiene	667	10 (1.5%)	20 (3%)	637 (95.5%)
Cough etiquette	667	2 (0.25%)	25 (3.75%)	640 (96%)
Surface disinfection	667	3 (0.5%)	113 (16.9%)	551 (82.6%)
Physical distancing	667	6 (0.9%)	216 (32.4%)	445 (66.7%)
Avoiding crowding place	667	11 (1.6%)	180 (27%)	476 (71.4%)
Participants with influenza-like illness (*N* = 352)				
Wearing facemask	352	100 (28.5%)	146 (41.5%)	106 (30.1%)
Hand hygiene	352	23 (6.5%)	187 (53.1%)	142 (40.4%)
Cough etiquette	352	70 (19.9%)	157 (44.6%)	125 (35.5%)
Surface disinfection	352	72 (20.5%)	150 (42.6%)	130 (36.9%)
Physical distancing	352	79 (22.4%)	178 (50.6%)	95 (27%)
Avoiding crowding place	352	71 (20.2%)	139 (39.5%)	142 (40.3%)

### Influenza-like illnesses and overall protective measure score

**Fig. 3:**
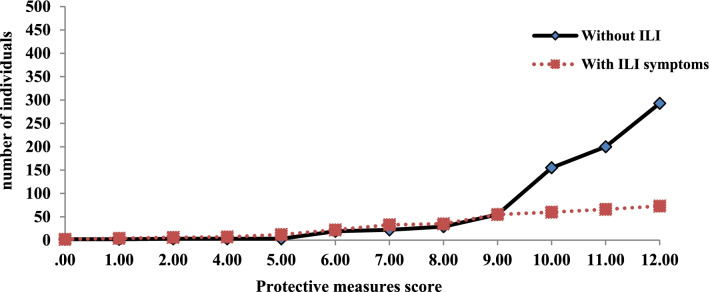
Personal protective measure score among Lebanese adults with and without symptoms of influenza-like illnesses

Figure 3 depicts the occurrence of influenza-like diseases as a function of the protective measure score. Adults who did not have influenza-like illnesses had a higher level of personal protection measures against COVID-19, indicating a relationship between the implementation of these measures and the occurrence of influenza-like illnesses cases. Of the Lebanese adults who experienced influenza-like illnesses, 67% (*n* = 236) of them had a personal protective score below the median (median = 11) while 33% (*n* = 116) had scores above the median. A significant difference was revealed in the bivariate analysis between individuals who scored below the median and those who scored above the median (χ^2^ = 33.87, *p* < 0.001, OR = 0.347).

### Factors associated with the occurrence of influenza-like illness

The first regression analysis that included covariates such as gender, age group, marital status, health status, educational level, current smoking status, and vaccination status in addition to the implementation of personal protective measures found that the baseline covariates did not significantly affect the occurrence of influenza-like illnesses except for influenza intake (*p* = 0.019). Therefore, the baseline covariates were removed from the regression model, and the analysis was rerun with the six protective measures after controlling the influenza vaccination status. As seen in Table 3, the final regression analysis showed that Lebanese adults who wore their facemasks frequently or always were less likely to suffer from symptoms of influenza-like illnesses than others who did wear the mask sometimes, rarely, or did not wear the facemask at all (aOR = 0.452, 95% CI = 0.349–0.693, *p* < 0.001). Similarly, adults who adopt the following protective measures frequently or always: washing hands (aOR = 0.608, 95% CI = 0.524–0.922, *p* < 0.001), respecting cough etiquette (aOR = 0.763, 95% CI = 0.598–0.918, *p* < 0.001), disinfecting surfaces (aOR = 0.892, 95% CI = 0.632–0.911, *p* = 0.012), avoiding crowded places (aOR = 0.739, 95% CI = 0.688–0.903, *p* = 0.049), respecting physical distancing (aOR = 0.646, 95% CI = 0.482–0.833, *p* = 0.031) were less likely to report symptoms of influenza-like illnesses when compared with those who did not adhere at all to these measures. Of note, no significant difference in terms of the occurrence of influenza-like illness between adults who did not adhere at all to the above-mentioned protecting measures and those who only applied these measures occasionally.

**Table 3:** Multivariable logistic regression of the factors associated with ILI among Lebanese adults

	*p *value	aOR	Confidence interval 95%	
			Lower	Upper
Wearing facemask				
Never/rarely	Ref.			
Sometimes	0.142	0.843	0.751	1.829
Frequently/always	< 0.001	0.452	0.349	0.693
Hand hygiene				
Never/rarely	Ref.			
Sometimes	0.072	0.903	0.767	2.012
Frequently/always	< 0.001	0.608	0.524	0.922
Cough etiquette				
Never/rarely	Ref.			
Sometimes	0.231	0.978	0.832	3.012
Frequently/always	< 0.001	0.763	0.598	0.918
Surface disinfection				
Never/rarely	Ref.			
Sometimes	0.389	0.832	0.724	1.571
Frequently/always	0.012	0.892	0.632	0.911
Physical distancing				
Never/rarely	Ref.			
Sometimes	0.128	0.805	0.601	2.129
Frequently/always	0.031	0.646	0.482	0.833
Avoiding crowding place				
Never/rarely	Ref.			
Sometimes	0.521	0.656	0.502	2.032
Frequently/always	0.049	0.739	0.688	0.903

## Discussion

The recent COVID-19 outbreak has stimulated a public-driven movement for prevention and governments worldwide have made many attempts to control the situation resulting from the emergence of COVID-19. This included the implementation of NPIs at personal and at country levels. Since other viral respiratory infections shared the same transmission dynamics with COVID-19 where these viruses spread through close contact, aerosols, and/or droplets, therefore, implemented measures could not only curtail the spread of SARS-CoV-2 but might also impact the occurrence of ILI and reduce the transmission of other viral respiratory infections.

### Study main findings

The current study argues that participants who exhibited a high level of adherence to COVID-19 personal protective measures were more likely of being free of influenza-like illnesses during the flu season compared to those who did not adhere to the measures. This strongly suggested that the protective measures taken against the spread of SARS-CoV-2 have also interrupted the spread of ILI. Our results were in line with the findings of previous studies that emphasized the potential of community-level strategies used to halt the spread of SARS-CoV-2 in lowering influenza transmission was revealed in several studies [16, 34, 35]. Furthermore, a longitudinal study conducted among international students found a twofold increased risk of ILI among students who did not implement all the personal protective measures compared with those who adhered to the measures [27]. Of note, a similar impact of implemented protective measures was detected in European countries such as Austria, Belgium, Italy, Germany, Spain, and the Netherlands [36].

Our finding revealed the potential of wearing facemasks regularly (frequently or always) in decreasing the occurrence of ILI among participants (aOR = 0.452) in comparison with other individuals who didn’t adhere to this protective measure. Based on the literature, several studies aimed to evaluate the effectiveness of facemask use in preventing pandemic influenza infection [37–45]. Some of the above-mentioned studies reported similar results as our study such as the findings of a cross-sectional survey conducted by Kim et al. [41] which revealed a significant protective effect of continuous mask use in children, relative to non-users (OR = 0.51; 95% CI 0.30–0.88), but a non-significant risk increase in irregular users relative to non-users (OR = 1.02; 95% CI 0.83–1.25). A cohort study conducted by Kuster et al. showed also that for each 10% increase in adherence to facial protection, there is a decrease (OR = 0.92) in the risk of being infected by influenza viruses among healthcare workers [44]. Similarly, Suess et al. found in their cluster randomized control trial a significantly protective effect of facemask use (OR = 0.28) [42]. However, some studies [37, 40, 45] found a non-significant protective effect of a facemask in preventing influenza infection.

In terms of hand hygiene, washing hands regularly and continually was found associated with a lower risk of suffering from ILI symptoms. Given the high compliance rate among study participants (95.5%) reporting always or frequently their hands, the results of this study provide a reliable estimate of the impact of hand hygiene in preventing influenza-like illnesses in the context of a pandemic. Our findings were consistent with the results of several studies evaluating the effectiveness of hand hygiene in preventing influenza infection [38, 39, 42–44, 46]. A study conducted by Aiello et al. reported that hand hygiene decreases respiratory infections by 16–21% [47]. Azor-Martinez et al. reported that the school absenteeism associated with pandemic influenza declined in schools that implemented hand sanitizer intervention [46]. Swess et al. found that the combination of hand hygiene with facemask use (OR = 0.26) was able to decrease the risk of secondary influenza infection [42]. However, inconsistent findings in terms of the protective effect of hand hygiene were reported by Kim et al. who found a non-significant protective effect of subjectively reported “frequent” hand-washing, with OR = 0.99 (95% CI 0.96–1.02). Of note, the protective effect of this intervention was more pronounced in studies where the frequency was defined objectively based on a minimum number of times individuals washed their hands daily.

Our study disclosed the potential of the adherence to physical distancing and avoidance of crowded places in decreasing the likelihood of experiencing symptoms of influenza-like illnesses when compared with those who did not adhere at all to these measures. Prem et al. reported the benefits of limiting social mixing [48]. However, a previous study conducted among international students did not find any significant association between cases of influenza-like illnesses and avoiding crowded places [27]. Of note, the evidence regarding the benefit of avoiding crowded places in preventing respiratory virus infection in individuals remains scarce.

Additionally, our study found a significant association between surface disinfection and reported cases of influenza-like illnesses among Lebanese adults. Our results were following a previous study that revealed the potential of surface disinfection effectively decreases secondary COVID-19 transmission in households [49].

In terms of cough etiquette which was found associated with a lower likelihood of suffering from ILI, no studies were found that evaluated the effectiveness of respiratory etiquette on ILI transmission. However, a study appraising the efficiency of cough etiquette in blocking aerosol particles, found that cough etiquette did not block the release or dispersion of aerosol droplets, particularly those smaller than one micron in size [50]. Of note, influenza particles are extremely small (0.08–0.12 μm in diameter) [51], and could easily be transmitted in small droplets expelled during sneezing or coughing.

It is noteworthy that influenza vaccine intake was found to decrease the likelihood of occurrence of ILI among participants, it is important to mention that the recommendations for vaccination against influenza during this influenza season did not change compared with previous seasons. In Lebanon, the influenza vaccines are normally administered from 1 October each year. However, in the 2020/2021 season, the influenza vaccination was delayed a little bit due to the delay in purchasing the influenza vaccines. However, it is unlikely that the vaccination could have resulted in the sudden sharp decline seen in the occurrence of ILI since the latest could result from other respiratory viruses. Of note, the low circulation of influenza viruses for one or two seasons shown in several countries could minimize the exposure of young children to these viruses and enlarge the group of children who will be susceptible in the following influenza season.

In summary, adherence to each of the six personal protective measures recommended by the WHO reduced cases of ILI among Lebanese adults participating in this study. In addition, a synergic implementation of all of the above-mentioned personal protective measures offered higher protection to individuals compared with single measures.

### Strengths of the study

To the best of our knowledge, the present study is the first in Lebanon to successfully explore the association between the practice of personal protection measures and the risk of influenza-like illnesses in the community during the 2020–2021 flu season. Given the current economic crisis, the fear of COVID-19, the overstrain on the healthcare system and the recommendation issued by the government to minimize face-to-face interaction, Lebanese adults with ILI could hesitate to visit healthcare services and often delay accessing healthcare. Thus, our findings provide a snapshot of the reported cases of ILI in the community where healthcare avoidance may be an important confounder affecting the findings of the healthcare-based acute respiratory infection surveillance system. Therefore, our participants could represent a category that may not be captured in hospital visits.

### Limitations

Several limitations should be acknowledged in this study. First, the cross-sectional design of our study precludes the ability to make a causal inference and its retrospective nature presents a risk of reporting biases, which could overestimate the true effectiveness of the personal protective measures in preventing ILI infection, as ILI cases and free ILI cases may misjudge their adoption of these measures to justify their infection status. For example, participants gave a self-reported history of ILI which could be subject to recall bias. In addition, respondents gave their self-evaluation using subjective terms to define the frequency of their implementation of personal protective measures against COVID-19 such as “rarely”, “occasionally” or “sometimes”, which may be affected by social desirability and could lead to a possible overestimation of their implementation. To resolve any potential social desirability bias, an anonymous online survey was used and participants were assured of the confidentiality of their responses in the introductory part of the survey. The study evaluated only the preventive effects of six personal protective measures; other measures may have contributed to suppressing transmission of respiratory infections such as lockdown, mass gathering cancellation, and school closure. While there are plenty of positive benefits to using online surveys for data collection, there are also some drawbacks that should be acknowledged in this study. For example, it was difficult to obtain a truly random sample of participants as they are limited to those that have subscripted to the internet service, and who are available at the time the researchers post the instrument. Therefore, sampling bias could have arisen in several ways as the questionnaire would only have reached persons who were downwind and would only have been completed by those who were literate and those who were sufficiently interested in the topic to take the time to respond. Despite weighing over gender, age, and geographical regions, selection bias might be present given the use of the convenience sampling technique. However, this is the best-case scenario as the sample is probably representative of the population. The convenience sampling technique used in our study limits the generalizability of our findings. However, a large sample was used to reduce the sampling error and to increase the study power. Lastly, a knowledge gap related to the appropriate “threshold” for an adequate personal protective measure scale, exists. This will likely vary based on individual factors such as exposure, susceptibility, and risk of adverse outcomes.

### Implications of the study and future directions

Our results indicate that implementing and complying with these measures can substantially help in minimizing the impact of other respiratory viruses and reducing their associated burden on healthcare systems. Such findings could be useful in designing prevention programs for respiratory infections in high risky settings (crowded places, entertainment venues, nursing homes.) where respiratory infections are very harmful and also emphasize the importance of implementing these measures even after the end of the current pandemic for vulnerable individuals such as elderly and immunocompromised. Another important implication of this study was the frequency in which these measures should be performed to curtail the transmission of COVID-19 and ILI, therefore, the general public is urged to actively comply with these preventative measures. Lastly, risk communication strategies to enhance the public’s knowledge in this area are crucial to clarify locations and situations where viral contact is likely and to emphasize the value of engaging in such protective behaviors.

## Conclusion

This study constitutes a contribution to the non-pharmaceutical interventions research in the context of a pandemic. Our findings highlighted the potential of personal protective measures against COVID-19 in reducing the transmission of ILI. The optimal intervention strategy may combine broad recommendations for frequent hand hygiene, combined with targeted facemask use and other protective measures among high-risk populations. Exploring trends detected by the national severe acute respiratory infection surveillance system is recommended to confirm the utility of these measures.

## Data Availability

Due to its proprietary nature, the datasets generated during the current study are not publicly available, but are available from the corresponding author on reasonable request with the permission of the Ministry of Public Health.

## Abbreviations

ILI: Influenza-like illnesses
COVID-19: Coronavirus disease 2019
aOR: Adjusted odds ratio
SPSS: Statistical Package for Social Sciences
NPI: Non-pharmaceutical intervention
CI: Confidence interval
SARS-CoV-2: Severe acute respiratory syndrome-corona virus-2
CVI: Content validity index
I-CVI: Item-level content validity index
S-CVI: Scale-level content validity index
